# Association Between Lifetime Hallucinogen Use and Valvular Heart Disease: Findings from the All of Us Research Program

**DOI:** 10.1080/02791072.2026.2673845

**Published:** 2026-05-18

**Authors:** Kevin H. Yang, Miranda Rasmussen, Kush Bhatt, Nora Satybaldiyeva, Wayne Kepner, Alison A. Moore, Jaclyn Bergstrom

**Affiliations:** a Department of Psychiatry, University of California San Diego School of Medicine, La Jolla, CA, USA;; b Stanford University School of Medicine, Palo Alto, CA, USA;; c Department of Medicine, Division of Geriatrics, Gerontology and Palliative Care, University of California San Diego School of Medicine, La Jolla, CA, USA

**Keywords:** All of Us Research Program, hallucinogen, LSD, MDMA, psilocybin, valvular heart disease

## Abstract

Recent literature suggests potential associations between hallucinogen use and valvular heart disease (VHD) due to prolonged activation of serotonin 5-HT_2B_ receptors, which may lead to valvular fibrosis – a condition also linked to drugs including fenfluramine and pergolide. Despite these concerns, epidemiological studies exploring this association are lacking. This exploratory analysis investigated associations between lifetime hallucinogen use and VHD using cross-sectional data from US adults with linked electronic health record data in the NIH All of Us Research Program who completed the Lifestyle survey. This survey included questions about lifetime hallucinogen use (lysergic acid diethylamide [LSD], mushrooms/psilocybin, 3,4-Methylenedioxymethamphetamine [MDMA]/ecstasy, ketamine, phencyclidine [PCP]). Multivariable logistic regression models examined the association between hallucinogen use and VHD, adjusting for sociodemographic factors and other confounding health conditions. Our sample comprised 286,842 adults (mean age 50.8 [SD 16.7], 61.4% female, 60.6% White). Among them, 13.2% reported lifetime hallucinogen use. Individuals with lifetime hallucinogen use had lower unadjusted VHD prevalence compared to those without lifetime hallucinogen use (3.6% vs. 4.7%, *p* < .001). However, after adjusting for confounders, models revealed modestly increased VHD odds (aOR = 1.08, 95% CI: 1.01–1.55, *p* = .017). This exploratory study found that hallucinogen use was associated with modestly increased VHD odds after adjustment, requiring confirmation through longitudinal research.

## Introduction

In recent years, there has been a surge in clinical research and public interest surrounding hallucinogens, including psilocybin, lysergic acid diethylamide (LSD), and 3,4-methylenedioxymethamphetamine (MDMA) (Hadar et al. 2023; Livne et al. 2022). This renewed interest is driven, in part, by promising clinical studies showing efficacy in treating various psychiatric conditions, leading to the United States (US) Food and Drug Administration (FDA) breakthrough designations for MDMA in post-traumatic stress disorder (2017), psilocybin in treatment-resistant depression (2018) and major depressive disorder (2019), and LSD in generalized anxiety disorder (2024) (Luoma et al. 2020; Yao et al. 2024). Concurrently, a wave of decriminalization efforts has increased the prevalence of hallucinogen use in the US (Keyes and Patrick 2023; Livne et al. 2022; Siegel et al. 2023; Yang et al. 2024), coinciding with diminishing perceptions of risk among the general population (Barnett et al. 2024; Livne et al. 2022).

As hallucinogen use continues to rise, establishing a comprehensive safety profile for these substances is increasingly important. Recent years have seen increases in emergency department visits and poison control cases related to hallucinogen use (Farah et al. 2024; Garel et al. 2024; Leas et al. 2024), with reported adverse effects ranging from acute cardiovascular and psychiatric symptoms to longer-term perceptual abnormalities (Evans et al. 2023; Hinkle et al. 2024; Marrocu et al. 2024; Myran et al. 2025; Simonsson et al. 2023, 2024; Yang, Han, and Palamar 2022; Yerubandi et al. 2024). One area of pharmacological interest is the theoretical relationship between hallucinogen use and valvular heart disease (VHD). This association stems from the pharmacologic action of many hallucinogens on serotonin receptors, specifically the 5-HT_2B_ receptor subtype (Nahlawi, Ptaszek, and Ruskin 2025; Rouaud, Calder, and Hasler 2024; Wsół 2023). The relationship between serotonergic drugs and VHD has been recognized since the 1960s, with prolonged activation of 5-HT_2B_ receptors in the heart linked to valvular fibrosis (Elangbam 2010). This association led to the withdrawal of several drugs from the US market, including fenfluramine for obesity in 1997 and pergolide for Parkinson’s disease in 2007 (Elangbam 2010; Rouaud, Calder, and Hasler 2024). Despite theoretical concerns in recent reviews (McIntyre 2023; Nahlawi, Ptaszek, and Ruskin 2025; Neumann et al. 2024; Rouaud, Calder, and Hasler 2024; Tagen et al. 2023; Wsół 2023), there is a notable lack of epidemiological research exploring this potential association. Furthermore, the growing popularity of microdosing (Yang et al. 2024), which involves taking sub-perceptual doses of psychedelics on a routine basis, raises additional concerns about chronic receptor activation that could lead to VHD (Rouaud, Calder, and Hasler 2024; Tagen et al. 2023). At the same time, however, there are several factors that may mitigate these theoretical concerns, including episodic rather than chronic administration of psychedelics in therapeutic contexts, as well as variations in receptor pharmacology (e.g., 5-HT_2B_ receptor affinity, functional selectivity, and receptor binding kinetics) across different hallucinogens (McIntyre 2023; Rouaud, Calder, and Hasler 2024). These pharmacological nuances highlight the complexity of predicting clinical outcomes and underscore the need for empirical investigation beyond theoretical considerations.

In June 2023, the FDA issued guidance recommending evaluation of the potential for drug-induced VHD associated with psychedelic substances (Office of the Commissioner 2024). However, examining this link has been challenging due to several factors, including high costs of echocardiography studies and limitations in existing databases (e.g., National Survey on Drug Use and Health, Monitoring the Future, National Health and Nutrition Examination, UK Biobank) that lack combined substance and cardiovascular outcome data (Barnett 2022). The All of Us Research Program uniquely addresses these gaps by combining self-reported hallucinogen use data with EHR-derived VHD diagnoses. In keeping with the FDA draft guidance, we conducted an exploratory analysis using data from the National Institutes of Health (NIH) All of Us Research Program to investigate this potential association. Given the nature of this analysis, our findings should be considered hypothesis-generating rather than confirmatory, especially given the inherent methodological limitations associated with the cross-sectional design and assessment of lifetime hallucinogen use through a single-item question encompassing multiple substances. Despite these constraints, this exploratory analysis offers important preliminary insights that can guide hypothesis development for more confirmatory research designs.

## Methods

### Data source

We analyzed cross-sectional data from the All of Us Research Program, an NIH nationwide research initiative designed to advance precision medicine by building one of the most diverse biomedical databases in history, aiming to enroll at least 1 million people. The All of Us Research Program collects a wide range of data from participants including survey data, electronic health record (EHR) data, and physical measurements. Participants can sign up directly through the NIH All of Us Research Program website or through participating health care provider organizations. At the time of our analysis, the program had enrolled 413,457 participants, with more than 80% from communities traditionally underrepresented in biomedical research, including those underrepresented by race and ethnicity, age, sexual orientation and gender identity, low income and educational attainment, rural residence, and disability. The All of Us Research Program is approved by the NIH Institutional Review Board (IRB). Participants sign an informed consent authorizing the collection of their data. Due to the secondary nature of this analysis and no direct involvement of human subjects, the University of California San Diego IRB exempted this study from review. The study followed Strengthening the Reporting of Observational Studies in Epidemiology (STROBE) reporting guidelines.

### Study sample

**Figure 1.**
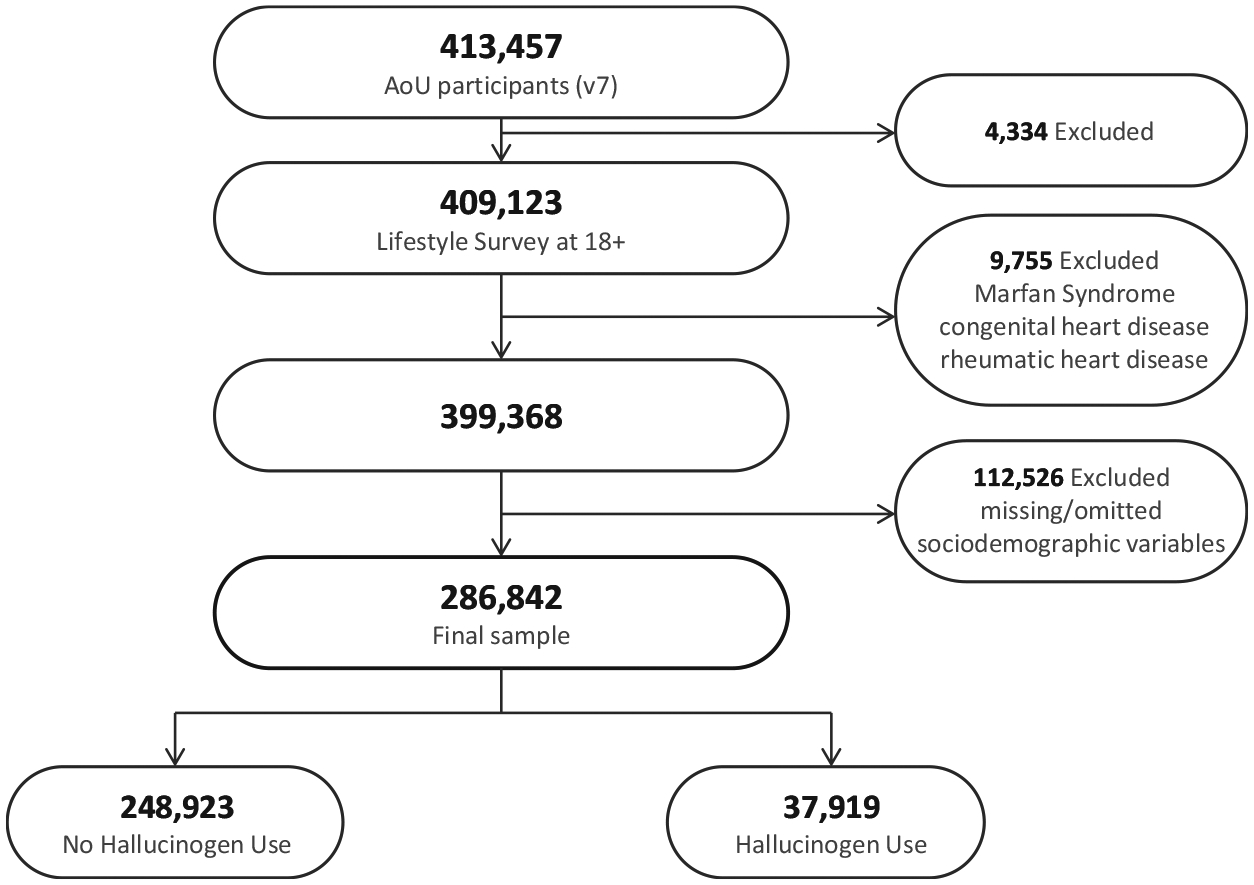
Participant flow chart of the study inclusion/exclusion criteria in the All of Us Research Program. Note: AoU = All of Us Research Program.

At the time of our analysis, 413,457 participants were enrolled in version 7 of the All of Us Research Program dataset. We included adults aged ≥18 who had completed the Lifestyle survey, yielding 409,123 participants (99.0% completion rate) and excluding 4,334 participants who did not meet this criterion. To mitigate preexisting condition bias, we further excluded 9,755 participants with congenital heart disease, rheumatic heart disease, or Marfan syndrome based on EHR diagnosis (Supplemental Table S1). We then removed participants who had missing or omitted sociodemographic variables (*n* = 112,526), yielding 286,842 participants in our final analytic sample. Figure 1 illustrates the participant flow and exclusion criteria.

## Measures

### Hallucinogen use

Lifetime hallucinogen use was assessed through the Lifestyle survey. Participants were asked: “In your LIFETIME, which of the following substances have you ever used?” with an option for hallucinogens defined as “LSD, acid, mushrooms, PCP, Special K, ecstasy, etc.”

### Valvular heart disease

We identified valvular heart disease using EHR data based on relevant SNOMED codes (Supplemental Table S1).

### Other substance use

Lifetime use of substances was assessed through the Lifestyle survey. Smoking status was determined by the question: “Have you smoked at least 100 cigarettes in your entire life?” Participants who answered “Yes” were classified as ever smokers, while those who answered “No” were classified as never smokers. The survey also assessed lifetime cannabis use and use of other substances. We combined other substances to include cocaine, methamphetamine, inhalants, sedatives, street opioids, prescription stimulant misuse, and prescription opioid misuse.

### Health conditions

We identified other health conditions using electronic health records based on relevant SNOMED codes (Supplemental Table S1), including hypertension, hyperlipidemia, diabetes, coronary arteriosclerosis, obesity, heart failure, lupus, and cardiac arrhythmia (any type). We identified and adjusted for these health conditions based on known risk factors for valvular heart disease, as established in previous literature (Coffey et al. 2021; Kubala et al. 2022; Lindman et al. 2016; Supino et al. 2006).

### Sociodemographic characteristics

Sociodemographic information was extracted from participants’ survey responses in the All of Us Basics survey. We included age (continuous variable), sex (male, female, other), race/ethnicity (Non-Hispanic [NH] White, NH Asian, NH Black, NH Other, Hispanic), marital status (married, divorced, never married, widowed), annual household income (<$10,000, $10,000–$24,999, $25,000–$34,999, $35,000–$49,999, $50,000–$74,999, $75,000–$99,999, $100,000–$149,999, $150,000–$199,999, ≥$200,000), employment status (employed, not employed), insurance status (insured, not insured), and education status (less than high school, high school, some college, college degree or higher).

### Statistical analyses

We employed descriptive statistics to compare sociodemographic characteristics, other substance use, and health conditions associated with valvular heart disease based on lifetime hallucinogen use status and reported *p*-values from t-tests and chi-squared tests. We then conducted a multivariable logistic regression analysis using a bidirectional stepwise model selection based on Akaike Information Criterion (AIC) to identify the best-fitting model. This model was used to examine the association between lifetime hallucinogen use and valvular heart disease. The AIC-based selection process removed lifetime cannabis use and lifetime use of other illicit substances from the final model (full model AIC: 77323; final model AIC: 77320), with the remaining covariates retained. Collinearity was assessed using adjusted Generalized Standard Error Inflation Factors (aGSIF); all values were below 1.4, indicating no concerning multicollinearity.

We reported adjusted odds ratios (aORs) with 95% confidence intervals (CIs) and corresponding *p*-values. Statistical significance was defined as *p* < .05. All analyses were performed using R (version 4.5.0; R Foundation for Statistical Computing, Vienna, Austria) in a Jupyter Notebook within the All of Us Workbench environment.

## Results

Of 286,842 participants included in the analysis, the mean (SD) age was 50.8 (16.7) years. The majority of the sample were female (61.4%), White (60.6%), married (53.0%), unemployed (50.2%), insured (93.9%), and college educated (50.4%), and the plurality had annual income <$10,000 (15.8%) (Table 1). Approximately one in eight participants (13.2%) reported lifetime hallucinogen use.

Compared to those without lifetime hallucinogen use, individuals reporting lifetime use were more likely to be younger, male, White, Non-Hispanic, divorced or never married, employed, and to have at least some college education (all *p* < .001). These individuals were also more likely to report other substance use, including smoking at least 100 cigarettes in their lifetime, lifetime cannabis use, and lifetime use of other illicit substances (all *p* < .001).

Regarding health conditions, individuals reporting lifetime hallucinogen use generally had lower prevalence rates compared to those without lifetime hallucinogen use. They had fewer health conditions, including valvular heart disease (3.6% vs. 4.7%, *p* < .001), as well as lower rates of hypertension, hyperlipidemia, obesity, diabetes, cardiac arrhythmia, coronary arteriosclerosis, heart failure, and lupus (all *p* < .001).

In the unadjusted analysis, lifetime hallucinogen use was associated with lower odds of valvular heart disease (OR = 0.76, 95% CI: 0.72–0.81, *p* < .001) (Table 2). However, after adjusting for potential confounders in the multivariable logistic regression model, the association between lifetime hallucinogen use and valvular heart disease reversed direction, indicating a modest but statistically significant association (aOR = 1.08, 95% CI: 1.01–1.55, *p* = .017) (Table 3).

Further analysis of the adjusted model revealed several factors associated with increased odds of valvular heart disease. Significantly higher odds were observed for increasing age (aOR = 1.25 per 10-year increase), sex (female, aOR = 1.53; other, aOR = 1.28; reference: male), never married status (aOR = 1.14; reference: married), higher income level ($100,000–$149,999, aOR = 1.09; $150,000-$199,999, aOR = 1.33; ≥$200,000, aOR = 1.40; reference: $50,000-$74,999), and college education (aOR = 1.14; reference: high school). Various health conditions were associated with increased odds of valvular heart disease, including cardiac arrhythmia (aOR = 3.42), heart failure (aOR = 2.74), hyperlipidemia (aOR = 2.34), hypertension (aOR = 2.28), coronary arteriosclerosis (aOR = 2.00), and lupus (aOR = 1.91) (all *p* < .001). Conversely, significantly lower odds of valvular heart disease were observed for lower-income categories (<$10,000, aOR = 0.86; $10,000-$24,999, aOR = 0.90; $25,000-$34,999, aOR = 0.89; reference: $50,000-$74,999), NH Black race (aOR = 0.89; reference: NH White), uninsured status (aOR = 0.59; reference: insured), lifetime smoking of at least 100 cigarettes (aOR = 0.90), and diabetes diagnosis (aOR = 0.89) (all *p* < .01).

## Discussion

In this large-scale, exploratory cross-sectional analysis of a diverse, nationwide cohort from the All of Us Research Program, we provide novel insights into the potential association between lifetime hallucinogen use and VHD. Our findings reveal a modest (i.e., small effect size; Chen, Cohen, and Chen 2010) but statistically significant association between self-reported lifetime hallucinogen use and EHR-derived VHD diagnosis, after adjusting for various sociodemographic factors and health conditions. To our knowledge, this is the first epidemiologic study evaluating these potential associations, warranting further investigation in light of the changing legal and clinical landscape surrounding hallucinogens and their increasing recreational use. It is crucial to emphasize that, based on the exploratory analysis of this project, these findings should be considered hypothesis-generating rather than confirmatory, and our results should be interpreted as preliminary evidence that warrants further investigation.

Our findings align with theoretical concerns raised in recent literature about potential associations between hallucinogen use and VHD (McIntyre 2023; Nahlawi, Ptaszek, and Ruskin 2025; Neumann et al. 2024; Rouaud, Calder, and Hasler 2024; Tagen et al. 2023; Wsół 2023). These concerns stem from various hallucinogens’ action on serotonin 5-HT_2B_ receptors, which has been linked to valvular fibrosis in previous studies of serotonergic drugs. This association was first observed with migraine treatments (methysergide, ergotamine) in the 1960s and has since expanded to include various drug classes such as appetite suppressants (fenfluramine, dexfenfluramine), dopamine agonists (pergolide, cabergoline), and recreational drugs like MDMA. For instance, Rothman et al. (2000) and Fitzgerald et al. (2000) independently reported that fenfluramine and its metabolite, norfenfluramine, drugs known to cause VHD, were potent agonists of 5-HT_2B_ receptors. Zanettini et al. (2007) conducted an echocardiographic prevalence study in patients taking dopamine agonists for Parkinson’s disease and found that moderate to severe regurgitation in any valve was more frequent in patients taking pergolide (23.4%) or cabergoline (28.6%) compared to controls (5.6%). Similarly, Droogmans et al. (2007) reported that 28% of MDMA users had abnormal echocardiographic results compared with none in the control group.

Of importance to note is the observed reversal in the direction of association between hallucinogen use and VHD – from a protective relationship in unadjusted analyses to a risk relationship after adjustment – which requires careful interpretation. This pattern likely reflects important confounding factors related to the demographic and health profiles of hallucinogen users in our sample; hallucinogen users in our sample were younger with fewer cardiovascular risk factors (e.g., hypertension, diabetes), naturally leading to lower unadjusted VHD rates. However, after statistically accounting for these differences in multivariable adjustment, we found a modest positive association between hallucinogen use and VHD. Thus, our adjusted findings suggest that lifetime hallucinogen use is associated with modestly increased, not decreased, odds of VHD. This reversal highlights that the relationship between hallucinogen use and VHD is complex and potentially confounded by numerous factors, reinforcing the need for more sophisticated longitudinal studies with detailed exposure assessments to better understand any potential causal relationship.

Although these drugs belong to various pharmacological classifications, the biological mechanism underlying this association is thought to involve the activation of 5-HT_2B_ receptors on heart valve leaflets, leading to proliferation of valve interstitial cells and excessive extracellular matrix production (Hutcheson et al. 2011). While some drugs like fenfluramine and pergolide were withdrawn from the US market due to these effects, the long-term impact of newer substances such as psychedelics remains unclear and warrants further investigation. It is important to note that different hallucinogens may have varying affinities for the 5-HT_2B_ receptor, which could result in differential risks for VHD. For example, psilocin binds to the 5-HT_2B_ receptor with greater affinity than pergolide, a drug with an established association with VHD (Rouaud, Calder, and Hasler 2024). Hallucinogens also have complex pharmacology involving multiple receptor systems beyond 5-HT_2B_, including other serotonin receptor subtypes and dopamine receptors, which may also influence cardiovascular outcomes and warrant consideration in future research (for additional reviews of receptor binding affinities and pharmacological considerations, see Nichols 2016; Tagen et al. 2023). Furthermore, recent studies have begun to evaluate the cardiovascular safety of psychedelic microdosing directly. Effinger et al. (2025) found no ventricular or valvular remodeling in mice after 8 weeks of LSD microdosing, noting that 5-HT_2B_ receptor activation was substantial but short-lived compared to cardiotoxins like *d*-fenfluramine. In the first clinical study to assess valvulopathy following repeated psychedelic administration in humans, Daldegan-Bueno et al. (2026) reported no significant cardiac abnormalities on echocardiography after 8 weeks of LSD microdosing. However, both studies were limited to LSD microdosing and short durations. Future pharmacological and clinical research should aim to elucidate these substance-specific risks, determine clinically relevant receptor occupancy thresholds, and evaluate longer-term cardiovascular effects across different hallucinogenic substances.

Our study also identified several other variables, including various health conditions, to be associated with VHD, which were largely consistent with existing literature. As expected, we found significantly increased odds of VHD among individuals with cardiac arrhythmia, heart failure, hyperlipidemia, hypertension, coronary arteriosclerosis, and lupus. These findings align with previous research identifying these conditions as risk factors for VHD (Coffey et al. 2021; Kubala et al. 2022; Lindman et al. 2016; Supino et al. 2006).

Interestingly, our analysis revealed an unexpected negative association between lifetime smoking of at least 100 cigarettes and VHD. This finding contrasts with established literature that generally associates smoking with increased cardiovascular risks (Coffey et al. 2021; Lindman et al. 2016; Supino et al. 2006). Several factors could explain this discrepancy. First, our measure of smoking ≥100 cigarettes may not adequately capture the nuances of smoking behavior, such as current smoking status, intensity, or duration. Second, there may be unmeasured confounding factors influencing this relationship. Third, potential survival bias may be at play, where smokers with VHD might have lower survival rates and thus be underrepresented in our sample. Finally, differences in healthcare-seeking behavior between smokers and nonsmokers could impact VHD diagnosis rates. This unexpected finding underscores the complexity of VHD etiology and highlights the need for more detailed, longitudinal studies for further clarification.

The association between hallucinogen use and VHD observed in our study, while modest, raises important considerations for clinical practice and public health. Healthcare providers should be aware of this potential risk, especially given the increasing prevalence of hallucinogen use. This awareness may inform patient screening practices, particularly for individuals who may be at elevated risk for adverse health outcomes, including those with a history of hallucinogen use disorder, frequent users, and individuals engaging in long-term microdosing (Rouaud, Calder, and Hasler 2024). The decision to explore this further, such as with echocardiography screening, should be made on an individual, case-by-case basis, considering the patient’s overall risk profile. From a public health perspective, our findings contribute to the ongoing discussion about the safety profile of hallucinogens. As several jurisdictions move toward decriminalization or legalization of certain psychedelics, policymakers should consider these potential risks in their regulatory frameworks. Regulatory frameworks should include monitoring systems for potential cardiovascular effects and education about possible risks, particularly for vulnerable populations.

## Limitations

This study has several important limitations to consider. First, our analysis relies on cross-sectional data, which precludes drawing causal inferences about the relationships between hallucinogen use and health outcomes. Longitudinal studies are needed to establish temporality and directionality of these associations.

Second, our measure of hallucinogen use was based on self-reported lifetime use, which does not capture important nuances such as frequency, recency, or type of hallucinogen used. While the All of Us dataset grouped LSD, psilocybin/mushrooms, MDMA/ecstasy, ketamine, and PCP together as hallucinogens, it is important to note that not all of these substances are known to activate 5-HT_2B_ receptors; specifically, ketamine and PCP are not associated with this mechanism of action. However, according to the 2022 National Survey on Drug Use and Health data, lifetime use of PCP (2.5%) and ketamine (1.8%) among adults aged ≥18 is considerably less prevalent than lifetime use of LSD (12.0%), psilocybin/mushrooms (12.3%), and MDMA/ecstasy (8.6%). This suggests that the potential impact of including ketamine and PCP in our hallucinogen category may be relatively minor compared to the more commonly used substances that do act on 5-HT_2B_ receptors. Nevertheless, this broad categorization may obscure differential effects of specific substances. Additionally, the definition of hallucinogen use as “LSD, acid, mushrooms, PCP, Special K, ecstasy, etc.” may introduce some ambiguity with its open-ended “etc.” designation. However, it is important to note that the survey separately asks about other substances such as cannabis, cocaine, methamphetamine, inhalants, sedatives, street opioids, and misuse of prescription medications in distinct categories within the same questionnaire, thus reducing the likelihood that participants would have included these other substances in their responses to the hallucinogen question.

Regarding grouping of hallucinogens and use of lifetime prevalence, it is worth noting that this approach is a common practice in epidemiological research on psychedelics given the scarcity of large-scale surveys that capture such data. For instance, numerous studies have employed lifetime hallucinogen and psychedelic use to investigate associations with a wide range of outcomes, including hypertension (Jones et al. 2022; Simonsson et al. 2021), heart disease (Simonsson et al. 2021), cancer (Barnett et al. 2022), substance use disorders (Jones, Lipson, and Nock 2022), depression (Jones and Nock 2022), suicidality (Hendricks et al. 2015), and criminal behavior (Hendricks et al. 2018).

Third, All of Us employs convenience sampling and intentionally oversamples communities historically underrepresented in biomedical research. While this approach enhances diversity, our findings may not be fully representative of the US population. Fourth, All of Us relies on survey data and EHR data, which may introduce potential biases; survey responses may be subject to recall bias or social desirability bias, while EHR data may contain misclassification or inconsistencies in diagnoses. Additionally, not all study participants completed the voluntary surveys or provided consent to link their data to EHR records, which may have introduced selection bias. Furthermore, our analytic sample excluded participants with missing sociodemographic data, which could introduce selection bias if missingness is not at random. Fifth, while we adjusted for a number of potential confounders, residual confounding remains a possibility. Unmeasured factors, such as substance use patterns or healthcare-seeking behaviors, could influence both hallucinogen use and health outcomes.

Our findings underscore the need for more comprehensive research into the potential associations between hallucinogen use and VHD. Future studies addressing the limitations of our work are needed, along with several other key approaches. Prospective longitudinal studies are needed to establish temporality and assess how different use patterns might affect VHD risk. Additionally, integrating clinical data, particularly echocardiography results, would provide more precise measurements of valvular structure and function. Finally, further investigation into the pharmacological properties of different hallucinogens and their interactions with 5-HT_2B_ receptors could help elucidate substance-specific risks. These multi-faceted approaches will be essential in developing a more comprehensive understanding of this potential relationship. Such research could ultimately inform clinical practice, public health policy, and harm reduction strategies in both recreational and therapeutic contexts.

## Conclusion

In conclusion, notwithstanding the limitations of our study, we detected a potential signal between hallucinogen use and VHD, warranting further and more thorough clinical research to better understand this potential association. Given the exploratory nature of the study, results should not be taken as confirmatory; rather, they should be used to inform hypothesis testing in well-controlled future studies. As both recreational and therapeutic use of hallucinogens continue to expand amid policy changes and clinical developments, ongoing research into their cardiovascular effects remains crucial for informed decision-making by individuals, clinicians, and policymakers alike.

## Supplementary Material

Supp 1

Supplemental data for this article can be accessed online at https://doi.org/10.1080/02791072.2026.2673845

## Figures and Tables

**Table 1.** Characteristics of participants in the All of Us Research Program, by lifetime hallucinogen use.

	Respondents, No. (%)			
Characteristic	Total sample	No lifetime hallucinogen use	Lifetime hallucinogen use	*p*-value
Total No. of participants	286,842	248,923	37,919	
Age, mean (SD), y	50.8 (16.7)	51.2 (16.8)	48.4 (15.6)	<0.001
Sex				<0.001
Male	108,686 (37.9)	89,904 (36.1)	18,782 (49.5)	
Female	176,069 (61.4)	157,168 (63.1)	18,901 (49.8)	
Other	2,087 (0.7)	1,851 (0.7)	236 (0.6)	
Race/Ethnicity				<0.001
NH White	173,808 (60.6)	144,661 (58.1)	29,147 (76.9)	
NH Black	48,140 (16.8)	45,928 (18.5)	2,212 (5.8)	
NH Asian	9,745 (3.4)	8,965 (3.6)	780 (2.1)	
NH Other	13,261 (4.6)	11,232 (4.5)	2,029 (5.4)	
Hispanic	41,888 (14.6)	38,137 (15.3)	3,751 (9.9)	
Marital Status				<0.001
Married	151,981 (53.0)	133,155 (53.5)	18,826 (49.6)	
Divorced	49,821 (17.4)	42,362 (17.0)	7,459 (19.7)	
Never married	71,535 (24.9)	60,968 (24.5)	10,567 (27.9)	
Widowed	13,505 (4.7)	12,438 (5.0)	1,067 (2.8)	
Income				<0.001
<$10,000	45,192 (15.8)	39,550 (15.9)	5,642 (14.9)	
$10,000-$24,999	39,829 (13.9)	34,349 (13.8)	5,480 (14.5)	
$25,000-$34,999	25,153 (8.8)	21,938 (8.8)	3,215 (8.5)	
$35,000-$49,999	28,326 (9.9)	24,627 (9.9)	3,699 (9.8)	
$50,000-$74,999	38,507 (13.4)	33,470 (13.4)	5,037 (13.3)	
$75,000-$99,999	30,270 (10.6)	26,236 (10.5)	4,034 (10.6)	
$100,000-$149,999	37,566 (13.1)	32,564 (13.1)	5,002 (13.2)	
$150,000-$199,999	17,698 (6.2)	15,287 (6.1)	2,411 (6.4)	
≥$200,000	24,301 (8.5)	20,902 (8.4)	3,399 (9.0)	
Employed	142,936 (49.8)	123,085 (49.4)	19,851 (52.4)	<0.001
Insurance				<0.001
Yes	269,378 (93.9)	233,827 (93.9)	35,551 (93.8)	
No	15,771 (5.5)	13,654 (5.5)	2,117 (5.6)	
Unknown	1,693 (0.6)	1,442 (0.6)	251 (0.7)	
Education				<0.001
<High school	19,160 (6.7)	17,651 (7.1)	1,509 (4.0)	
High school	47,613 (16.6)	42,163 (16.9)	5,450 (14.4)	
Some college	75,584 (26.4)	64,385 (25.9)	11,199 (29.5)	
College graduate	144,485 (50.4)	124,724 (50.1)	19,761 (52.1)	
Substance use, lifetime				
Smoke ≥100 cigarettes	115,226 (40.2)	90,052 (36.2)	25,174 (66.4)	<0.001
Cannabis	155,524 (54.2)	118,175 (47.5)	37,349 (98.5)	<0.001
Other illicit substances^1^	87,471 (30.5)	55,984 (22.5)	31,487 (83.0)	<0.001
Health condition				
Valvular Heart Disease	13,053 (4.6)	11,683 (4.7)	1,370 (3.6)	<0.001
Hypertension	78,313 (27.3)	70,373 (28.3)	7,940 (20.9)	<0.001
Hyperlipidemia	74,141 (25.8)	66,285 (26.6)	7,856 (20.7)	<0.001
Obesity	52,187 (18.2)	47,311 (19.0)	4,876 (12.9)	<0.001
Diabetes	35,620 (12.4)	32,533 (13.1)	3,087 (8.1)	<0.001
Cardiac arrhythmia	32,138 (11.2)	28,579 (11.5)	3,559 (9.4)	<0.001
Coronary arteriosclerosis	20,030 (7.0)	17,872 (7.2)	2,158 (5.7)	<0.001
Heart failure	11,593 (4.0)	10,440 (4.2)	1,153 (3.0)	<0.001
Lupus	2,547 (0.9)	2,317 (0.9)	230 (0.6)	<0.001

Note: SD = standard deviation. NH = Non-Hispanic.

1 Other illicit substances includes cocaine, methamphetamine, inhalants, sedatives, street opioids, prescription stimulant misuse, and prescription opioid misuse.

**Table 2.** Univariate odds of valvular heart disease in the All of Us Research Program.

Characteristic	OR (95% CI)	*p*-value
Hallucinogen use	0.76 (0.72–0.81)	<.001
Age^1^	1.78 (1.75–1.80)	<.001
Sex		<.001
Male	1 [Reference]	
Female	0.87 (0.84–0.90)	
Other	1.33 (1.10–1.58)	
Race		<.001
NH White	1 [Reference]	
NH Black	0.69 (0.66–0.73)	
NH Asian	0.40 (0.34–0.45)	
NH Other	0.80 (0.73–0.87)	
Hispanic	0.55 (0.52–0.59)	
Marital Status		<.001
Married	1 [Reference]	
Divorced	1.10 (1.05–1.15)	
Never Married	0.54 (0.52–0.57)	
Widowed	2.02 (1.90–2.15)	
Income		<.001
<$10,000	0.61 (0.57–0.65)	
$10,000-$24,999	1.02 (0.95–1.08)	
$25,000-$34,999	0.86 (0.79–0.92)	
$35,000-$49,999	0.91 (0.85–0.98)	
$50,000-$74,999	1 [Reference]	
$75,000-$99,999	1.00 (0.93–1.07)	
$100,000-$149,999	0.95 (0.89–1.02)	
$150,000-$199,999	1.00 (0.91–1.08)	
≥$200,000	1.04 (0.96–1.12)	
Employed		<.001
Yes	1 [Reference]	
No	2.17 (2.09–2.26)	
Insurance		<.001
Yes	1 [Reference]	
No	0.23 (0.19–0.26)	
Unknown	0.24 (0.14–0.36)	
Education		<.001
<High school	0.93 (0.86–1.02)	
High school	1 [Reference]	
Some college	1.13 (1.07–1.20)	
College graduate	1.17 (1.11–1.23)	
Substance use, lifetime		
Smoke ≥100 cigarettes	1.25 (1.21–1.30)	<.001
Cannabis	0.74 (0.71–0.77)	<.001
Other illicit substances^2^	0.87 (0.84–0.90)	<.001
Health condition		
Hypertension	9.55 (9.16–9.95)	<.001
Hyperlipidemia	10.33 (9.92–10.77)	<.001
Obesity	3.43 (3.31–3.55)	<.001
Diabetes	4.08 (3.93–4.24)	<.001
Cardiac arrhythmia	12.70 (12.24–13.18)	<.001
Coronary Arteriosclerosis	4.08 (3.93–4.24)	<.001
Heart Failure	13.88 (13.29–14.51)	<.001
Lupus	3.89 (3.48–4.33)	<.001

Note: OR = odds ratio. CI = confidence interval. NH = Non-Hispanic.

1 10-year unit.

2 Other illicit substances includes cocaine, methamphetamine, inhalants, sedatives, street opioids, prescription stimulant misuse, and prescription opioid misuse.

**Table 3.** Multivariable^1^ odds of valvular heart disease in the All of Us Research Program.

	aOR (95% CI)	*p*-value
Hallucinogen use	1.08 (1.01–1.55)	.017
Age^2^	1.25 (1.23–1.28)	<.001
Sex		<.001
Male	1 [Reference]	
Female	1.53 (1.46–1.59)	
Other	1.28 (1.04–1.55)	
Race		.002
NH White	1 [Reference]	
NH Black	0.89 (0.83–0.94)	
NH Asian	0.87 (0.74–1.00)	
NH Other	0.98 (0.89–1.08)	
Hispanic	0.94 (0.87–1.01)	
Marital Status		.004
Married	1 [Reference]	
Divorced	1.01 (0.96–1.07)	
Never Married	1.14 (1.07–1.21)	
Widowed	1.00 (0.93–1.08)	
Income		<.001
<$10,000	0.86 (0.79–0.95)	
$10,000-$24,999	0.90 (0.83–0.98)	
$25,000-$34,999	0.89 (0.82–0.97)	
$35,000-$49,999	0.94 (0.86–1.01)	
$50,000-$74,999	1 [Reference]	
$75,000-$99,999	1.06 (0.98–1.15)	
$100,000-$149,999	1.09 (1.02–1.18)	
$150,000-$199,999	1.33 (1.21–1.46)	
≥$200,000	1.40 (1.28–1.52)	
Employed		.14
Yes	1 [Reference]	
No	1.04 (0.99–1.09)	
Insurance		<.001
Yes	1 [Reference]	
No	0.59 (0.50–0.69)	
Unknown	0.74 (0.45–1.15)	
Education		<.001
<High school	0.93 (0.85–1.03)	
High school	1 [Reference]	
Some college	1.01 (0.94–1.07)	
College graduate	1.14 (1.07–1.22)	
Smoke ≥100 cigarettes	0.90 (0.86–0.94)	<.001
Health condition		
Hypertension	2.28 (2.16–2.42)	<.001
Hyperlipidemia	2.34 (2.22–2.47)	<.001
Obesity	1.04 (0.99–1.09)	.13
Diabetes	0.89 (0.85–0.94)	<.001
Cardiac arrhythmia	3.42 (3.28–3.58)	<.001
Coronary Arteriosclerosis	2.00 (1.90–2.10)	<.001
Heart Failure	2.74 (2.60–2.90)	<.001
Lupus	1.91 (1.68–2.17)	<.001

Note: aOR = adjusted odds ratio. CI = confidence interval. NH = Non-Hispanic.

1 The multivariable model was selected using Akaike Information Criterion (AIC); this process removed lifetime cannabis use and lifetime use of other illicit substances from the final model. The association between hallucinogen use and VHD was consistent in the full model (aOR = 1.08, 95% CI: 1.00–1.16, *p* = .044) and final model (aOR = 1.08, 95% CI: 1.01–1.55, *p* = .017).

2 10-year unit.

## Data Availability

Data can be accessed via the All of Us Research Workbench (researchallofus.org). Information on data analysis is available from the authors upon request.
