# Supplementary material for: Association Between Lifetime Hallucinogen Use and Valvular Heart Disease: Findings from the All of Us Research Program
Source: J Psychoactive Drugs. Author manuscript; Available in PMC 2026 May 29. (PMC13220840; doi:10.1080/02791072.2026.2673845)
Supplement: Supp 1 [file NIHMS2178964-supplement-Supp_1.docx]

**Supplemental Table 1.** Systematized Nomenclature of Medicine (SNOMED) codes for disease identification in the All of Us Research Program.

| **Variable** | **SNOMED Code** |
| --- | --- |
| **Included** |  |
| Valvular Heart Disease | 368009 |
| Hypertension | 38341003 |
| Hyperlipidemia | 55822004 |
| Diabetes | 73211009 |
| Coronary Arteriosclerosis | 53741008 |
| Obesity | 414916001 |
| Heart Failure | 84114007 |
| Lupus | 200936003 |
| Cardiac arrhythmia | 698247007 |
| **Excluded** |  |
| Congenital Heart Disease | 13213009 |
| Marfan syndrome | 19346006 |
| Rheumatic Heart Disease | 23685000 |

**Note:** SNOMED = Systematized Nomenclature of Medicine.
